# Assessing a Novel Thyroid-Stimulating Antibody Bioassay as a Predictor of Radioactive Iodine Therapy Efficacy in Graves' Disease

**DOI:** 10.7759/cureus.76698

**Published:** 2024-12-31

**Authors:** Seigo Tachibana, Yuji Nagayama, Takashi Fukuda, Kento Katsuyama, Daisuke Tatsushima, Yusuke Mori, Hisakazu Shindo, Hiroshi Takahashi, Shinya Sato, Hiroyuki Yamashita

**Affiliations:** 1 Department of Endocrinology, Yamashita Thyroid Hospital, Fukuoka, JPN; 2 Department of Surgery, Yamashita Thyroid Hospital, Fukuoka, JPN

**Keywords:** graves’ disease, predictive index, radioactive iodine therapy, rait, thyroid-stimulating antibody bioassay, tsab bioassay

## Abstract

Objective: No studies have reported the use of the Biosensor TSAb assay (TSAb (Bio)) for predicting the therapeutic efficacy of radioactive iodine therapy (RAIT) for Graves' disease (GD). Therefore, we evaluated the usefulness of this novel thyroid-stimulating antibody (TSAb) bioassay kit, which uses a cyclic adenosine monophosphate (cAMP) biosensor, in predicting the therapeutic efficacy of RAIT in 20 patients with GD.

Methods: We defined the RAIT outcome using two criteria: (i) thyroid weight reduction to <8 g and (ii) thyroid weight reduction to >80% at 12 months post-RAIT. For criteria (i) and (ii), regression analysis was performed for thyroid-stimulating hormone (TSH) receptor autoantibody (TRAb), TSAb (Bio), TSAb (Bio)/TRAb, I-131 absorbed dose/TSAb (Bio), and I-131 absorbed dose/(TSAb (Bio)/TRAb). After evaluating the significance of each factor, receiver operating characteristic (ROC) analysis was also performed.

Results: Among the five aforementioned anti-TSH receptor antibody parameters, only I-131 absorbed dose/(TSAb (Bio)/TRAb) showed statistical significance in the multivariate analysis (p = 0.004 and p = 0.021 for each criterion). Although I-131 absorbed dose/(TSAb (Bio)/TRAb) correlated significantly with the thyroid weight (r = -0.54, p = 0.015), it did not correlate with thyroid weight reduction rates at 12 months post-RAIT. ROC analysis also demonstrated a good predictive value (area under the curve of approximately 0.8 with a cut-off value of 3.9) for I-131 absorbed dose/(TSAb (Bio)/TRAb) in predicting thyroid weight at 12 months post-RAIT.

Conclusions: TSAb (Bio) is a useful predictor of RAIT efficacy when evaluated as I-131 absorbed dose/(TSAb (Bio)/TRAb). This index was defined by dividing a factor that positively affects RAIT outcomes (I-131 absorbed dose) by a factor negatively affecting it (i.e., (TSAb (Bio)/TRAb)). The therapeutic efficacy of RAIT can be expected with an I-131 absorbed dose/(TSAb (Bio)/TRAb) value of ≥3.9. Adjusting the I-131 dosage based on this index may lead to more effective treatment outcomes.

## Introduction

The conventional thyroid-stimulating antibody (TSAb) assay is based on enzyme immunoassay (EIA), referred to here as TSAb (EIA). Still, a rapid, simplified assay method has been desired owing to the time-consuming nature and multiple procedures required by EIA. A new TSAb bioassay system, the Biosensor TSAb assay (referred to here as TSAb (Bio)), was recently developed. This bioassay uses a human embryonic kidney cell line 293, genetically engineered to express the human thyroid-stimulating hormone (TSH) receptor and a cAMP-dependent luminescent biosensor [1]. The Japanese Ministry of Health, Labour and Welfare insured TSAb (Bio) in 2022. Compared with the conventional TSAb assay, TSAb (Bio) offers a shorter assay time and a simpler procedure and has shown excellent diagnostic accuracy for Graves' disease (GD) and high sensitivity to thyroid eye disease [2,3]. Consequently, TSAb (Bio) has become the mainstay of TSAb measurement in Japan. While both the conventional TSAb assay (TSAb (EIA) and TSH receptor autoantibody (TRAb) assay have previously been shown to be useful in predicting the therapeutic efficacy of radioactive iodine therapy (RAIT) for GD [4-7], no studies have reported the use of TSAb (Bio) for this purpose. Among the many factors reported to be useful predictors of RAIT therapeutic effect for GD - such as thyroid weight, radioactive iodine uptake (RAIU), thyroid weight reduction post-treatment, TRAb, TSAb (EIA), free T4 (FT4), and thyroid peroxidase antibodies [4-12] - factors measurable before RAIT treatment are particularly valuable for the prediction. In this context, the TSAb assay is crucial. Therefore, we aimed to determine whether TSAb (Bio) is useful in predicting RAIT therapeutic efficacy.

## Materials and methods

Ethics statements

This retrospective study was conducted in accordance with the tenets of the Declaration of Helsinki and was approved by the Ethics Committee of Yamashita Thyroid Hospital (approval number: 2024-3). All patients provided consent for the use of their clinical data through opt-out on the website.

Participants

Out of 122 patients with GD who underwent RAIT at our hospital between July 2016 and October 2023, 24 consented to the use of their stored sera for research purposes. RAIT was performed in nine cases due to adverse effects of antithyroid drug (ATD) treatment, in 12 cases due to resistance to ATD treatment, and in three cases due to recurrence after subtotal thyroidectomy. Cases of GD recurrence following subtotal thyroidectomy were excluded from this study owing to the lack of information on the remaining thyroid gland, timing of recurrence, and treatment details, as the surgery occurred 20-30 years ago. Additionally, one case with a dose of I-131 per thyroid weight of ≤100 µCi/g was removed [13], resulting in a total of 20 cases included in this study.

Radioactive iodine therapy (RAIT)

Iodine restriction was performed seven days before RAIT, and ATD and potassium iodide (KI) were withdrawn four days before RAIT. Three-hour RAIU values (RAIU-3hr) were evaluated with 123-I the day before RAIT and 24-hour RAIU values (RAIU-24hr) on the day of RAIT. After the administration of I-131, iodine restriction and ATD/KI withdrawal were continued for three days. The administered I-131 radiation dose was calculated from RAIU and thyroid weight, using the following formula: dose (μCi/gram (g)) = oral therapeutic I-131 dose given (mCi) × estimated 24 h RAIU (%) × 10/thyroid weight (g). I-131 absorbed doses (Gy) were determined using the Marinelli-Quimby equation, with an effective half-life of 5.7 days following the Manual for the Proper Use of Internal Therapy Using Radioiodide (I-131) Sodium Capsules issued by the Japanese Society of Nuclear Medicine and others.

Measurements and calculations

TSH, FT4, free triiodothyronine (FT3), and TRAb (third generation) were measured using Eclucys kits (Roche Diagnostics, Penzberg, Germany), and TSAb (Bio) was measured with a Biosensor TSAb kit (Yamasa Corporation, Choshi, Japan). Thyroid volumes were calculated by estimating both lobes and isthmus of the thyroid glands as rotational ellipsoids. They were used as thyroid weights, assuming a specific gravity of thyroid glands of approximately 1.0, as previously reported [14]. Thyroid weight reduction rates at 12 months post-RAIT were calculated using the following formula and expressed as a percentage:

Reduction rate = ([pre-RAIT thyroid weight - thyroid weight at 12 months post-RAIT] / pre-RAIT thyroid weight) × 100.

The RAIT treatment effect was assessed on the basis of thyroid weights and reduction rates in thyroid weights at 12 months post-RAIT rather than thyroid function at 12 months post-RAIT, as nine out of 20 patients were still on block and replacement therapy to avoid fluctuations in thyroid hormone levels after RAIT, hampering the assessment based on hormone levels. On the basis of previous data [6], two criteria for the efficacy of RAIT in GD were established: (i) thyroid weight of <8 g and (ii) thyroid weight reduction rate of >80% at 12 months post-RIAT.

Various parameters related to anti-TSH receptor antibody assays (TRAb, TSAb (Bio), TSAb (Bio)/TRAb, and I-131 absorbed dose/TSAb (Bio), and I-131 absorbed dose/[TSAb (Bio)/TRAb]) were analyzed using regression analysis between two groups with and without a treatment effect (the response group (R group) and non-response group (NR group)) for criterion 1 and %R group and %NR group for the criterion 2 (referred to previously and referred to in the following, respectively). In addition to TRAb and TSAb (Bio), three new indices were included: TSAb (Bio)/TRAb, I-131 absorbed dose/TSAb (Bio), and I-131 absorbed dose/(TSAb (Bio)/TRAb). TSAb (Bio)/TRAb represents relative TSAb activity compared to TRAb activity, that is, TSAb activity per 1 U/L TRAb. The indices I-131 absorbed dose/TSAb (Bio) and I-131 absorbed dose/((TSAb (Bio)/TRAb) were calculated by dividing a factor that positively affects RAIT outcomes (i.e., I-131 absorbed dose [11,15]) by a factor that negatively affects it (i.e., TSAb (Bio) and TSAb (Bio)/TRAb, respectively). Receiver operating characteristic (ROC) analysis was performed after evaluating the efficacy of each predictive factor.

Statistical analysis

Data are presented as mean ± standard deviation. Univariate analysis was performed using the Wilcoxon rank-sum test, and multivariate analysis was performed using multiple regression using a stepwise method. To evaluate multicollinearity, the variance inflation factor (VIF) was calculated before using the stepwise method. The VIF values for the variables included in the stepwise method ranged from 1.01 to 2.40, confirming that multicollinearity was not a significant issue. Correlations were evaluated using the Pearson correlation coefficient test. All analyses, including ROC curve analyses, were performed using JMP software (version 17.0; SAS Institute Inc., Cary, NC).

## Results

The clinical and biochemical parameters of the patients are shown in Table 1. The size of the thyroid glands, one of the criteria used to assess treatment efficacy, decreased to <8 g in 11 of 20 cases (the R group) but not in the other nine cases (the NR group) at 12 months post-RAIT. Univariate and multivariate analyses were performed to identify which antibody parameters (TRAb, TSAb (Bio), TSAb (Bio)/TRAb, I-131 absorbed dose/TSAb (Bio), and I-131 absorbed dose/(TSAb (Bio)/TRAb)) are useful for estimating RAIT outcomes. The I-131 absorbed dose itself was also analyzed as a control. As shown in Table 2, TSAb (Bio)/TRAb was significantly lower, and I-131 absorbed dose and I-131 absorbed dose/(TSAb (Bio)/TRAb) were significantly higher in the R group than in the NR group in the univariate analysis. There were no significant differences between the two groups regarding TRAb, TSAb (Bio), or I-131 absorbed dose/TASb (Bio). In the multivariate analysis using the stepwise regression method, only the difference in I-131 absorbed dose/(TSAb (Bio)/TRAb) remained significant.

**Table 1:** Summary of patients’ clinical and biochemical parameters. ^1^ATD, antithyroid drug; ^2^MMI, methimazole; ^3^RAIU, radioactive iodine uptake; ^4^TSH, thyroid stimulating hormone; ^5^FT4, free thyroxine; ^6^FT3, free triiodothyronine; ^7^RAIT, radioactive iodine treatment

Clinical and biochemical parameter	
Age (years)	40.2±15.8
Sex (male:female)	5:15
Treatment periods of Graves’ disease (months)	74.5±86.9
Durations of ATD^1^ administration (months)	51.0±52.1
Durations of MMI^2^ administration (months)	32.9±40.4
RAIU^3^-3hr (%)	44.7±20.0
RAIU-24hr (%)	63.0±16.1
131-I dose per unit weight of thyroid based on RAIU-3hr (μCi/g)	121.7±37.6
131-I dose per unit weight of thyroid based on RAIU-24hr (μCi/g)	184.5±59.3
131-I absorbed dose (Gy)	154.6±49.7
TSH^4^ (μU/mL)	0.37±0.87
FT4^5^ (ng/dL)	2.24±1.50
FT3^6^ (pg/mL)	8.91±7.57
Thyroid weights (g)	31.0±14.1
Drugs administered before RAIT^7^ (MMI:PTU:KI)	8:3:9
Thyroid weights at 12 months post-RAIT (g)	8.9±5.8
Reduction rates in thyroid weight at 12 months post-RAIT (%)	71.1±12.8

**Table 2:** Results of the univariate and multivariate analyses between the thyroid-stimulating hormone receptor-related parameter (TRAb, TSAb (Bio), TSAB (Bio)/TRAb, and 131-I absorbed dose/(TSAb (Bio)/TRAb)) and 131-I absorbed dose, and the efficacy of radioactive iodine treatment (RAIT) judged by <8 g thyroid weights at 12 months post-RAIT. ^1^R group, responder group with thyroid weights of <8 g at 12 months post-RAIT, ^2^NR group, non-responder group with thyroid weights of >8 g at 12 months post-RAIT, ^3^TRAb, thyrotrophin receptor antibody; ^4^TSAb, thyroid stimulating antibody; ^5^, significant in the univariate analysis; ^6^, significant in the multivariate analysis

	R group^1^ (n=11)	NR group^2^ (n=9)	p-value
TRAb^3^ (IU/L)	25.5±37.8	20.6±41.2	0.270
TSAb^4^ (Bio) (%)	1318.9±1682.1	2561.8±4337.4	0.732
TSAb (Bio)/TRAb	78.8±69.8	164.9±125.4	0.037^5^
131-I absorbed dose (Gy)	175.3±57.5	129.3±20,6	0.021^5^
131-I absorbed dose (Gy)/TSAb (Bio)	0.45±0.41	0.47±0.47	0.732
131-I absorbed dose /(TSAb (Bio)/TRAb)	3.6±1.9	1.3±1.1	0.014^5^, 0.004^6^

For the other criteria for assessing treatment efficacy, thyroid gland sizes were reduced by >80% in six patients (the %R group) but not in the remaining 14 patients (the %NR group). TRAb was significantly higher, TSAb (Bio)/TRAb was significantly lower, and I-131 absorbed dose and I-131 absorbed dose/(TSAb (Bio)/TRAb) were significantly higher in the %R group than in the %NR group in the univariate analysis. However, in the multivariate analysis, only I-131 absorbed dose/(TSAb (Bio)/TRAb) remained significant (Table 3). Therefore, only the I-131 absorbed dose/(TSAb (Bio)/TRAb) was significant in both analyses. All six patients in the %R group were also included in the R group, while in the other five patients, pretreatment thyroid weights averaged 18.6 ± 5.7 g with thyroid weight reduction rates of 69.87 ± 4.82%.

**Table 3:** Results of the univariate and multivariate analyses between the thyroid-stimulating hormone receptor-related parameter (TRAb, TSAb (Bio), TSAb (Bio)/TRAb, and 131-I absorbed dose/(TSAb (Bio)/TRAb)) and 131-I absorbed dose, and the efficacy of radioactive iodine treatment (RAIT) judged by >80% reduction of thyroid weights at 12 months post-RAIT. ^1^%R group, responder group with thyroid weight reductions of >80% at 12 months post-RAIT, ^2^%NR group, non-responder group with thyroid weight reductions of <80% at 12 months post-RAIT, ^3^TRAb, thyrotrophin receptor antibody; ^4^TSAb, thyroid stimulating antibody; ^5^significant in the univariate analysis; ^6^significant in the multivariate analysis

	%R group^1^ (n=6)	%NR group^2^ (n=14)	*p*-value
TRAb^3^ (IU/L)	39.4±47.1	16.4±33.6	0.026^5^
TSAb^4^ (Bio) (%)	2069.0±2023.2	1796.4±3569.8	0.248
TSAb (Bio)/TRAb	56.4±42.7	143.7±114.4	0.039^5^
131-I absorbed dose (Gy)	181.7±78.7	143.0±27.3	0.410
131-I absorbed dose (Gy)/TSAb (Bio)	0.29±0.32	0.53±0.46	0.364
131-I absorbed dose /(TSAb (Bio)/TRAb)	4.1±1.6	2.0±1.8	0.039^5^, 0.021^6^

**Figure 1:**
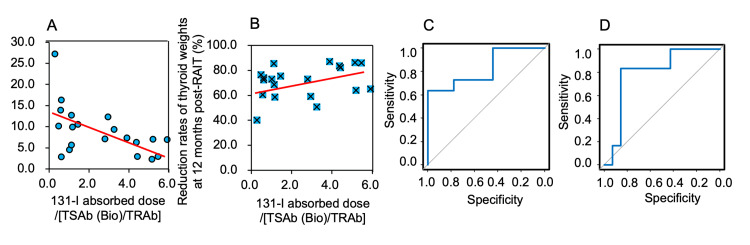
Results of correlations and receiver operating characteristic analyses. (A and B) Correlations between thyroid weights (A) or thyroid weight reduction rates (B) at 12 months post-RAIT and 131-I absorbed dose/(TSAb (Bio)/TRAb). (C and D) Receiver operating characteristic analyses of the 131-I absorbed dose/(TSAb (Bio)/TRAb) as a predictive factor for achieving <8 g thyroid weights (C) and >80% thyroid weight reduction (D), respectively.

A significant correlation of I-131 absorbed dose/(TSAb (Bio)/TRAb) was observed only with thyroid weights at 12 months post-RAIT (r = -0.54, p = 0.0149) and not with the thyroid weight reduction rates at 12 months post-RAIT (r = 0.32, p = 0.1645) (Figures 1A-1B), possibly due, in part, to the smaller number of patients in the %R group than in the R group (n = 6 vs. 11).

ROC curves were calculated for each parameter to evaluate the predictive value of I-131 absorbed dose/(TSAb (Bio)/TRAb), which was found to correlate with thyroid weights at 12 months post-RAIT and the reduction rates of thyroid weights at 12 months post-RAIT. For thyroid weights, at 12 months post-RAIT, the area under the curve (AUC) of I-131 absorbed dose/(TSAb (Bio)/TRAb) was 0.828 (95% confidence interval (CI): 0.642-1.000). The cut-off value, determined at the highest points of sensitivity and specificity, was set at 3.89, with a sensitivity of 63.6%, specificity of 100%, positive likelihood ratio of infinity, and negative likelihood ratio of 0.3636 according to the Youden index (Figure 1C). The same analysis for thyroid weight reduction rates at 12 months post-RAIT revealed an AUC of 0.798 (95% CI: 0.577-1.000) and a cut-off value of 3.89, with a sensitivity of 83.3%, specificity of 85.7%, positive likelihood ratio of 5.8314, and negative likelihood ratio of 1.1666 (Figure 1D).

## Discussion

Previous studies have shown that thyroid weights and their reduction rates are useful in predicting the efficacy of RAIT in GD [6,8,9]. All cases of hypothyroidism observed at 12 months post-RAIT had a thyroid volume of <8 mL [6]; the average rate of reduction of thyroid glands in hypothyroid and euthyroid cases at 12 months post-RAIT was 80.7 and 83.7%, respectively [6]. Additionally, most patients with thyroid reduction rates of ≥50% at three months post-RAIT developed hypothyroidism after RAIT [9]. Therefore, thyroid weights and their reduction rates at 12 months post-RAIT can indicate thyroid function after RAIT. On the basis of these reports, we chose to adopt thyroid weights and their reduction rates at 12 months post-RAIT as criteria for determining RAIT treatment efficacy, setting cut-off values of <8 g for thyroid weight and >80% for the reduction rate [6].

Our results indicate that TRAb, TSAb (Bio), and TSAb (Bio)/TRAb are ineffective predictors of RAIT therapeutic efficacy. Previous studies have reported inconsistent data regarding the usefulness of TRAb [4,5,16], likely because TRAb reflects the combined activity of stimulating and blocking (TBAb) antibodies [17,18]. The observed difference in the usefulness of TSAb (Bio) and TSAb (EIA) [6,7] may be attributed to the different measurement methods used for very high titers of TSAb between the two assays: dilution of homogenized cell solution of porcine thyroid cells in the TSAb (EIA) assay versus dilution of serum in the TSAb (Bio) assay. As aforementioned, Graves’ serum contains both TSAb and TBAb [17], and the TBAb assay is more affected by dilution since high antibody titers are required for TBAb to exert its inhibitory effect [19,20]. TSAb (Bio)/TRAb represents the stimulating activity per 1 IU/L of TRAb and has been suggested as a potential biomarker that correlates with orbital MRI findings in cases of thyroid eye disease [21]. Therefore, we hypothesized that TSAb (Bio)/TRAb might be a better indicator for predicting RAIT efficacy than TSAb alone. However, the differences in TSAb (Bio)/TRAb between the responder and non-responder groups did not reach statistical significance in our study.

A novel finding of this study is that the new index, I-131 absorbed dose/(TSAb (Bio)/TRAb), effectively estimates the therapeutic outcome of RAIT before treatment. The I-131 absorbed dose, calculated using factors affecting RAIT therapeutic efficacy, such as I-131 dose, RAIU, and thyroid weight, is positively correlated with RAIT efficacy [22-24], whereas TASb and TSAb (Bio)/TRAb may serve as biomarkers for resistance to RAIT. Therefore, this new index - calculated by dividing the I-131 absorption dose by TSAb (Bio)/TRAb, that is, dividing the factors affecting RAIT treatment positively from those negatively affecting it - is a strong theoretical predictor, as supported by our study’s findings.

In the ROC analysis for I-131 absorbed dose/(TSAb (Bio)/TRAb) using thyroid weights and thyroid weight reduction rates, the optimal cut-off value was 3.9. A particularly high positive likelihood ratio indicates that sufficient thyroid weight reduction can be expected when the cut-off value is ≥3.9. However, this index may not be sufficient for predicting RAIT inefficacy due to its low negative likelihood ratio, possibly because factors associated with treatment resistance, such as the duration of ATD administration [11,25], are not included. Considering these points, we believe this index is a valuable indicator for predicting RAIT treatment response.

Of interest, in addition to TSAb/TRAb [21], a new method for calculating thyroid-stimulation blocking antibody (TSBAb) activities (the blocking index) has been reported [26], which is calculated by dividing conventional TSBAb activities by the stimulation index (the value obtained with sample and buffer solution divided by the value with normal control and buffer solution). The development of such formulas has enabled a more accurate evaluation of antibody activities, and we examined the usefulness of similar formulas in this study and could demonstrate the usefulness of the new formula, the absorbed dose/(TSAb (Bio)/TRAb), for the prediction of RAIT efficacy.

This study has some limitations, including the inability to use thyroid hormone levels as markers of RAIT outcome, the lack of a direct comparison between the usefulness of TSAb (EIA) and TSAb (Bio), and the small sample size. While the stepwise method used for multivariate analysis in this study is a useful approach for variable selection, the results of this method are highly dependent on the dataset, which may compromise the generalizability of the model. This concern is particularly relevant when the sample size is small (20 cases in this study). Therefore, further studies with larger sample sizes are needed to confirm causal relationships.

## Conclusions

TSAb (Bio) is a useful predictor of RAIT treatment when evaluated alongside absorbed dose and TRAb. An index of I-131 absorbed dose/(TSAb (Bio)/TRAb) of ≥3.9 is expected to indicate effective RAIT. Adjusting the I-131 dosage based on this index may lead to more effective therapeutic outcomes.
